# Emotion-Aware Contextual Modelling for Robust Driver Fatigue Detection

**DOI:** 10.3390/s26134120

**Published:** 2026-06-30

**Authors:** Sebastian Budzan, Roman Wyżgolik

**Affiliations:** Department of Measurements and Control Systems, Silesian University of Technology, Akademicka 16, 44-100 Gliwice, Poland; roman.wyzgolik@polsl.pl

**Keywords:** fatigued driver detection, emotion-aware modelling, multimodal analysis, fatigue risk assessment, behavioural analysis

## Abstract

Vision-based driver fatigue detection remains challenging because facial signals associated with fatigue are often ambiguous, while geometric indicators such as Eye Aspect Ratio (EAR) and Percentage of Eye Closure (PERCLOS) are prone to false positives caused by normal facial activity, including smiling or speaking. This paper proposes a context-aware framework that integrates behavioural, geometric, and emotional information for robust fatigue assessment. Facial landmarks are extracted using MediaPipe Face Mesh, while adaptive eye-closure detection is performed through multi-stage validation combining EAR trajectories, mouth activity, head-pose analysis, and event-level filtering. Emotion recognition is achieved using an EfficientNet-B0 convolutional neural network trained on the AffectNet dataset, enabling frame-level estimation of facial expression probabilities. These predictions are aggregated into descriptors representing emotional variability and fatigue-related emotional relevance over time. Behavioural information obtained from blinking, yawning, head nodding, and validated PERCLOS is fused with emotional context to construct a multi-level fatigue assessment model. The final Driver Fatigue Risk Index combines physiological eye-closure information with contextual behavioural–emotional analysis, providing an interpretable estimation of driver state rather than a binary classification alone. Experimental evaluation on the NTHU-DDD dataset achieved 94% accuracy and demonstrated improved robustness under non-frontal head poses and expressive facial behaviour.

## 1. Introduction

Reports from organizations such as the U.S. National Highway Traffic Safety Administration, the AAA Foundation, the National Institutes of Health, and the National Sleep Foundation clearly indicate the threat posed by fatigued drivers. Due to the increasing number of vehicles and modern lifestyle factors, driver fatigue has become a significant contributor to road accidents. Studies show that fatigue is widely perceived as a common cause of accidents across countries [1], with 69% of respondents in the United States and 74% in Europe supporting this view. Furthermore, an estimated 17.6% of all fatal crashes between 2017 and 2021 involved drowsy driving [2], highlighting the need for effective driver support systems. In response, fatigue detection has become an important component of modern intelligent vehicle systems. It is also addressed within regulatory frameworks such as the EU General Safety Regulation (GSR), where fatigue monitoring is implemented in Driver Drowsiness and Attention Warning (DDAW) and Advanced Driver Distraction Warning (ADDW) systems.

Driver fatigue is a complex phenomenon that can manifest in multiple forms and originate from different causes, as described in [3]. It is commonly classified into active fatigue, resulting from prolonged task engagement, passive fatigue, associated with monotonous driving conditions, and sleepiness, which represents the most direct and critical form of fatigue. One of the earliest and least noticeable symptoms is microsleep, characterized by brief and often unintentional eye closures that are difficult to detect visually.

As fatigue progresses, more observable behavioural symptoms begin to appear. These include frequent yawning, repeated eye rubbing, touching the face, and difficulty maintaining eye opening and visual focus. Drivers may also exhibit changes in posture, such as reduced ability to keep the head upright, as well as increased variability in head movements. In later stages, fatigue can also affect communication patterns, leading to reduced interaction with passengers and overall behavioural withdrawal.

The combination of these gradual physiological and behavioural changes highlights the inherently multimodal nature of driver fatigue. As a result, numerous approaches have been proposed to quantify these symptoms using diverse sensing modalities and analytical techniques, including methods based on behavioural observation, physiological signals, and computer vision. Existing works emphasize different aspects of this problem, such as the role of individual driver characteristics in fatigue modelling [4], systematic classification of detection methods [5], and challenges related to real-world deployment and robustness [6]. In particular, significant attention has been devoted to vision-based behavioural analysis, especially methods focusing on the eye region [7], where fatigue-related symptoms such as blink frequency or eyelid closure can be directly quantified. In recent years, these approaches have been further advanced through deep learning techniques, particularly convolutional neural networks, which enable more robust feature extraction from facial data [8,9,10]. Beyond purely behavioural cues, recent approaches also incorporate higher-level contextual information, such as driver emotions [11] and distraction [12], either as complementary signals or within multimodal frameworks.

The need for robust feature representation and context-aware fusion is also supported by recent developments in related computer vision tasks. Robust human parsing studies have shown that high-resolution representations, auxiliary guidance, and boundary-aware modelling can improve feature stability in complex scenes [13]. Similarly, mask-guided visible–infrared fusion methods demonstrate that guided integration of complementary information sources can enhance robustness and representation quality [14]. Although these works address different application domains, they provide useful methodological motivation for the proposed framework, which combines geometric, behavioural, and emotional information in a context-aware and interpretable manner.

Although some recent approaches employ temporal deep-learning architectures such as 3D CNNs or Sequential Neural Networks, many methods still depend on frame-level geometric features and provide limited interpretability of temporal fatigue dynamics. In particular, classical approaches based on EAR, Mouth Aspect Ratio (MAR), or PERCLOS do not account for the ambiguity of facial expressions, where similar geometric patterns may arise from both fatigue and normal facial activity such as speaking or smiling. Furthermore, emotion-based methods are typically applied at the frame level and remain sensitive to prediction noise and dataset-specific characteristics. Consequently, there is a need for a framework that integrates temporal, behavioural, and emotional information in a more structured and interpretable manner.

The proposed framework combines several complementary components designed to improve the robustness and interpretability of vision-based driver fatigue detection. First, facial landmarks extracted using MediaPipe Face Mesh are used to derive geometric features related to eye openness, mouth activity, and head pose. Next, adaptive eye-closure detection and multi-stage event validation are applied to distinguish fatigue-related eye behaviour from normal facial activity such as smiling or speaking. In parallel, a convolutional neural network-based emotion recognition module estimates frame-level facial expression probabilities, which are subsequently aggregated over temporal windows to capture emotional variability and fatigue-related emotional context. The framework further integrates behavioural indicators, including blinking, yawning, nodding, and validated PERCLOS, within a unified behavioural–emotional fusion strategy. Finally, the proposed Driver Fatigue Risk Index provides a multi-level and interpretable assessment of driver state by combining physiological eye-closure information with temporally validated behavioural and emotional analysis.

The main contributions and novelties of this work are as follows:

- Introduction of a context-aware fatigue detection framework that integrates behavioural, geometric, and emotional information instead of relying on direct threshold-based interpretation.
- Development of an adaptive eye-closure detection and PERCLOS validation mechanism, including event-level filtering based on facial expression context (e.g., happy-related activity), reducing false positives caused by normal facial behaviour.
- Proposal of structured emotional descriptors, namely the Emotional Entropy Score (EES) and Emotion Weighted Score (EWS), capturing the temporal variability and fatigue relevance of facial expressions.
- Definition of the Behavioural–Emotional Fatigue Index (BEFI), enabling robust fusion of behavioural signals with emotional context in a unified formulation.
- Introduction of the Driver Fatigue Risk Index (DFRI) as a multi-level and interpretable fatigue assessment metric that combines physiological and contextual factors using thresholds defined at the index level rather than optimized directly for a single dataset.

The paper is organized as follows. Section 2 reviews related work, and Section 3 presents the proposed framework, including feature extraction, context-aware validation, emotional modelling, and the formulation of the BEFI and DFRI. Section 4 evaluates the method using datasets and case studies, and Section 5 concludes the paper.

## 2. Related Works

A wide range of approaches have been proposed for driver fatigue detection, employing signals derived from the vehicle, the driver’s physiology, and observable behaviour.

Vehicle-based fatigue detection methods rely on signals such as steering wheel movements, driver posture, and lane departure indicators. These approaches are attractive due to their non-intrusive nature and ability to provide continuous real-time monitoring without direct observation of the driver. Recent studies focus on long-term analysis of steering behaviour. For example, Li et al. [15] showed that variability in steering wheel grip force correlates with driver fatigue, while Lu et al. [16] proposed integrating surface electromyography (sEMG) sensors into the steering wheel to capture physiological signals in a non-invasive manner. Despite these advantages, such methods have limitations. Vehicle-based indicators are indirect measures of fatigue and can be affected by external factors, such as road conditions or individual driving style, reducing reliability. Moreover, approaches like sEMG require specialized hardware and increase system complexity, limiting practical applicability. Therefore, they are often insufficient as standalone solutions and motivate the use of complementary modalities.

Physiological approaches rely on signals such as EEG, ECG, and EMG, as well as pulse or respiration [17,18,19]. These methods provide accurate and direct measurements of fatigue but require contact-based sensors, which limits their practicality. In addition, they are often intrusive, may affect driver comfort, and require careful sensor placement and calibration, which complicates their deployment in real-world conditions. For instance, Arefnezhad et al. [20] proposed a dynamical modelling framework based on EEG signals to estimate driver sleepiness, identifying biomarkers correlated with increasing fatigue levels. In [21], the authors propose a solution that integrates multiple data modalities, including electroencephalogram (EEG) signals, electrocardiogram (ECG) signals, and facial images.

Behavioural approaches based on image analysis have become one of the most widely adopted and extensively studied solutions for driver fatigue detection. These methods rely on facial features, particularly from the eye and mouth regions, to estimate behavioural indicators such as blink frequency, eye closure duration, and yawning. Classical approaches use geometric descriptors and threshold-based metrics such as Eye Aspect Ratio (EAR) and Mouth Aspect Ratio (MAR) [22], while more advanced methods incorporate full-face representations with handcrafted features (e.g., HOG, LBP) and machine learning classifiers [23]. Other approaches focus on specific fatigue symptoms, such as yawning detection, using techniques such as Support Vector Machines (SVM) and Circular Hough Transform (CHT) [24]. These methods are commonly evaluated on publicly available datasets, including NTHU-DDD [25] and YawDD [26], which provide controlled scenarios for fatigue assessment. However, despite their effectiveness, behavioural approaches have several inherent limitations. First, many methods rely on fixed thresholds [27], which are highly sensitive to inter-subject variability and lighting conditions, reducing generalization. Second, feature-based approaches and handcrafted descriptors [28] often struggle to capture complex facial dynamics and may be sensitive to head pose variations. Third, methods focusing on single symptoms, such as yawning [29,30], are particularly prone to false detections caused by normal facial activity, such as speaking or smiling. Finally, most of these approaches operate at the frame level, lacking temporal context, which makes them sensitive to prediction noise and short-term fluctuations unrelated to fatigue. Moreover, these methods often suffer from limited robustness in real-world conditions, including illumination variations, occlusions (e.g., glasses or hands), and changes in camera viewpoint.

Recent approaches to driver fatigue detection increasingly rely on deep learning techniques applied to facial image analysis. These methods typically use visual features extracted from the eye and mouth regions, such as blink frequency, yawning, MAR, and eye closure duration, often combined with convolutional neural networks [31]. Some works have focused on improving robustness by accounting for individual differences—for example, You et al. [32] proposed a cascaded CNN architecture combined with landmark-based EAR estimation to better handle variations in eye size. Other approaches integrate advanced detection pipelines such as YOLOv5-based face detection with transformer-based classifiers [33], aiming to improve performance in complex scenarios. Additionally, the introduction of realistic datasets and temporal modelling, such as UTA-RLDD [34], has enabled the development of methods that incorporate sequential information rather than relying on single frames. Despite these advancements, several limitations remain. Many deep learning approaches still rely on frame-level predictions, making them sensitive to noise and short-term variations. Methods based on landmarks or predefined metrics (e.g., EAR) remain affected by head pose, occlusions, and individual differences. Additionally, complex models such as CNNs and transformers require significant computational resources, limiting real-time deployment. Although temporal modelling has been introduced [35], it is often limited to simple accumulation and does not explicitly distinguish between fatigue-related patterns and transient facial activity.

Recent studies increasingly highlight the role of affective processes in the evolution of driver fatigue. Experimental and physiological evidence suggests that fatigue is associated with systematic changes in emotional state, including an increase in negative affect and a reduction in positive emotions, particularly under conditions of sleep deprivation or prolonged cognitive load [36,37]. This indicates that emotions are not only correlated with fatigue, but may also provide valuable contextual information for its assessment. Moreover, fatigue-related emotional changes are often accompanied by altered attention patterns and increased susceptibility to distraction, further affecting driver behaviour and safety.

Motivated by these observations, recent approaches have begun to incorporate emotional information into fatigue detection frameworks. For example, Shang et al. [38] proposed a time-series fusion method that combines facial emotion recognition with behavioural indicators to jointly estimate driver fatigue and emotional state. Similar attempts can also be found in integrated driver monitoring systems, where emotional cues are analysed alongside fatigue- and distraction-related signals to improve situational awareness.

However, such approaches remain relatively limited in the literature. This is largely due to the inherent challenges associated with emotion recognition in real-world driving conditions, including high variability, ambiguity of facial expressions, and sensitivity to noise and environmental factors. Moreover, emotional states do not directly correspond to fatigue levels, making their interpretation less straightforward compared to that of classical physiological or behavioural indicators. Furthermore, these approaches often lack higher-level contextual understanding of emotional dynamics, making it difficult to distinguish between fatigue-related emotional patterns and transient or unrelated affective states.

As a consequence, existing methods typically treat emotional information as an auxiliary signal and rely on direct aggregation of features, without explicitly modelling their temporal variability or semantic relevance. In contrast, the approach proposed in this work introduces a set of structured descriptors and indices, including the Emotional Entropy Score (EES), Emotion Weighted Score (EWS), and Behavioural Drowsiness Score (BDS), which are further combined within the Behavioural–Emotional Fatigue Index (BEFI) and the Driver Fatigue Risk Index (DFRI). These components are designed to capture both the temporal stability and fatigue-related significance of emotional dynamics, enabling a more interpretable and robust integration of emotional context within fatigue detection beyond conventional score-level fusion methods, representing a novel step toward systematic modelling of emotional dynamics in driver fatigue detection.

## 3. Materials and Methods

### 3.1. Overview of the Proposed Framework

The proposed method assesses driver fatigue through a multi-stage pipeline that integrates behavioural and emotional information within a unified framework.

Although deep learning-based approaches can classify eye closure, mouth states, and drowsiness-related facial patterns directly from image sequences, relying exclusively on such models has several limitations in this application. Firstly, deep learning methods usually require large, diverse annotated datasets covering different camera angles, lighting conditions, subjects’ appearance, facial expressions and levels of fatigue. In driver drowsiness datasets, particularly those based on simulated or acted fatigue, there is a risk that the model will learn dataset-specific visual shortcuts, such as exaggerated mouth opening or characteristic facial gestures, rather than robust physiological indicators of fatigue. Secondly, purely data-driven models often lack interpretability, making it difficult to determine whether a decision was caused by eyelid closure, yawning, head pose, facial expression or another visual cue. This is particularly problematic in safety-critical driver monitoring applications, where the source of the fatigue decision must be traceable. Thirdly, unless sufficient variability is represented during training, deep learning models may be sensitive to domain shifts caused by changes in viewpoint, illumination, occlusion, the presence of glasses, changes in facial morphology, or non-frontal head poses.

For this reason, the proposed framework intentionally utilizes geometric descriptors such as EAR and Mouth Aspect Ratio (MAR). These features provide physically meaningful and interpretable measurements of eye openness and mouth activity. However, they are not used as simple fixed-threshold decision rules. Instead, EAR and MAR are treated as input signals for adaptive thresholding, temporal event validation, head-pose-related consistency analysis, and contextual behavioural–emotional modelling. This design combines the transparency of classical measurements with additional robustness against false positives caused by smiling, speaking, laughter, pose variation, or temporary landmark instability.

First, the input video is processed frame-by-frame to extract facial landmarks using MediaPipe Face Mesh, version 0.10.5, which is then used to compute geometric features such as EAR, MAR, and head pose.

**Figure 1:**
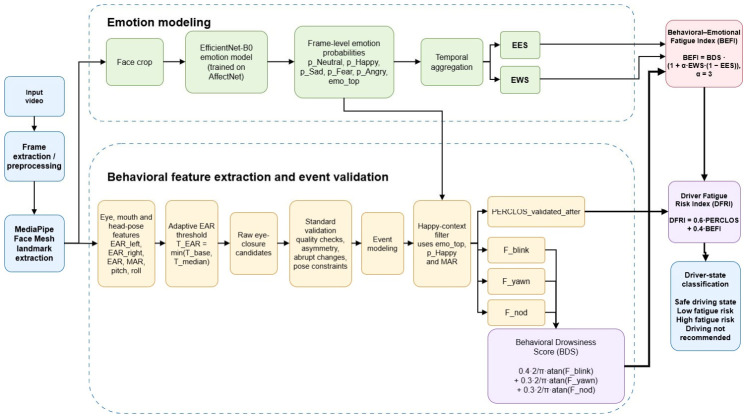
Scheme diagram of the proposed framework.

Candidate eye-closure events are then detected based on these signals using an adaptive EAR threshold and refined through a multi-stage validation procedure that includes quality checks and event-level modelling. To reduce false positives, a context-aware filtering mechanism is applied where low EAR events are interpreted alongside facial expression cues (e.g., a happy expression and mouth activity). In parallel, an emotion recognition model processes face crops to generate emotion probabilities at the frame level; these are then aggregated over time to create structured descriptors, i.e., the Emotional Entropy Score (EES) and the Emotion Weighted Score (EWS). These descriptors capture the variability and fatigue relevance of facial expressions. Behavioural signals, including validated PERCLOS and event frequencies (e.g., blinking, yawning, nodding), are combined to create the Behavioural Drowsiness Score (BDS). Behavioural and emotional information are then fused within the Behavioural–Emotional Fatigue Index (BEFI), which is integrated with PERCLOS to compute the Driver Fatigue Risk Index (DFRI). The final output is a multi-level classification of the driver’s state, providing a robust and interpretable assessment of fatigue risk. Although feature extraction is performed at the frame level, the proposed framework operates on temporally aggregated windows and event-level modelling, including continuous eye-closure episodes, temporal emotional descriptors, and behavioural frequency estimation. The scheme diagram is presented in Figure 1. In driver fatigue detection, one of the key processing stages is the reliable estimation of eye openness. This is commonly performed using facial landmarks, from which the EAR indicator is computed. EAR describes the ratio between the vertical eye opening and the eye width. A decrease in this value may indicate eyelid narrowing or eye closure, while a sustained low EAR is used to detect eye-closure events and to compute PERCLOS.

The classical dlib-based approach uses a sparse facial landmark model comprising 68 points, with six landmarks representing each eye. While this method is computationally efficient and performs well under controlled conditions, such as frontal face orientation and stable illumination, its main limitation is the small number of points used to describe the eye region. Since EAR is computed from only six landmarks, even minor localisation errors can significantly distort the result. This issue is particularly evident in non-frontal views, where perspective effects, partial visibility of the eyes, and unstable landmark detection can lead to EAR values that do not reflect actual eyelid behaviour.

The use of MediaPipe Face Mesh represents the face using a denser landmark mesh, while still allowing EAR computation from a standard six-point subset embedded in a more complete facial representation. This improves the stability of eye-region localisation under head rotation, perspective changes, and partial eye visibility, making the MediaPipe more effective than sparse landmark models.

For this reason, MediaPipe is a more suitable choice for analysing driver recordings where the camera is not perfectly frontal. Such conditions are common in real or semi-controlled setups, with cameras placed laterally, above, or below the face, and drivers not always looking directly ahead. Under these conditions, a denser landmark representation provides a more reliable EAR signal, improving eye-closure detection, PERCLOS estimation, and overall driver-state assessment.

However, MediaPipe does not eliminate all sources of error, as EAR still depends on factors such as image quality, illumination, facial expression, and head pose. In the proposed method, the use of MediaPipe is therefore justified by three main arguments. First, the denser facial representation improves the stability of landmark localisation around the eyes. Second, the method is better suited to moderate head rotation and non-frontal face orientation. Third, a more reliable EAR trajectory provides a better basis for subsequent processing stages, including eye-closure candidate detection, PERCLOS correction, facial-expression-context filtering, and final driver-state classification.

**Figure 2:**
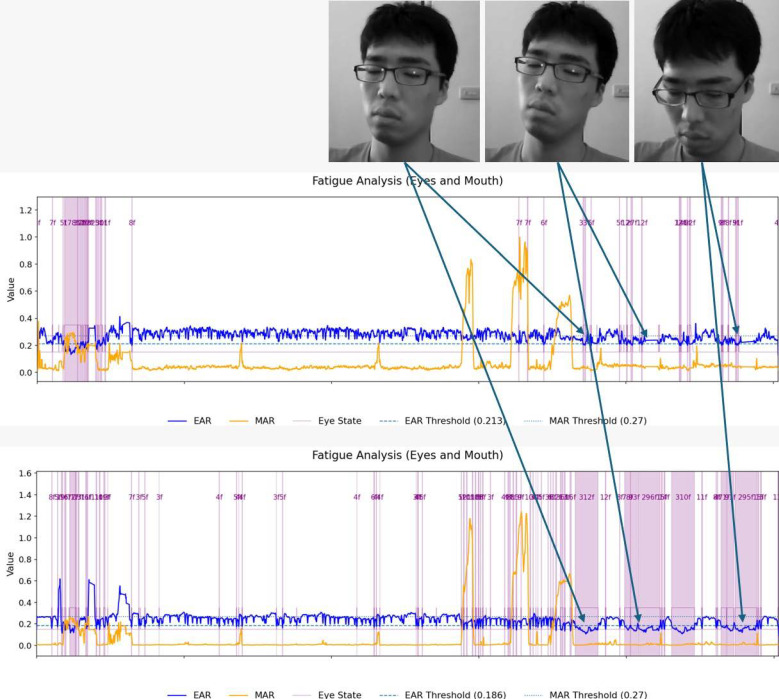
Comparison of the fatigue analysis for video sequence analysed using dlib and MediaPipe: selected video frames are shown at the top, EAR and MAR trajectories obtained with dlib in the middle, and EAR and MAR trajectories obtained with MediaPipe at the bottom.

Figure 2 illustrates three characteristic fragments of the video sequence created from the NTHU-DDD dataset for a selected person. In the analysed sequence, the subject (person) is slightly angled relative to the camera. The upper panel presents selected frames in which the subject has narrowed or closed eyes, including cases in which eyelid narrowing co-occurs with yawning. The middle panel shows the EAR and MAR trajectories obtained with dlib, whereas the lower panel shows the corresponding trajectories obtained with MediaPipe. The vertical arrows indicate corresponding moments in the video frames and in the signal plots.

In the dlib-based analysis, EAR values remain relatively high or change in a way that is weakly related to the visually observed eyelid narrowing. As a result, an algorithm relying on this signal may incorrectly estimate the number of eye-closure episodes and underestimate or distort PERCLOS. In the MediaPipe-based analysis, EAR decreases are more consistent with visual inspection: at the moments of eyelid narrowing, the signal drops more clearly relative to the baseline level. This indicates that MediaPipe better captures the local eyelid geometry under non-ideal face orientation relative to the camera.

It should be emphasised that the observed difference does not result from a different definition of the EAR indicator itself. In both cases, EAR is based on the ratio between vertical eye opening and eye width. The difference is instead related to the quality and density of the landmark representation. Dlib uses a small number of points around the eye, so the final value is strongly affected by the localisation of individual landmarks. MediaPipe Face Mesh provides a denser facial representation, and the selected eyelid landmarks are embedded in a more complete description of face geometry. As a result, EAR measurement may be more robust to factors such as moderate head rotation, partially side-view acquisition, and local facial-expression changes.

### 3.2. Frame-Level Feature Extraction and Context-Validated Event Modelling

Behavioural signals are not directly derived from frame-level thresholds but are constructed through a multi-stage process combining feature extraction, temporal aggregation, and context-aware validation. The Behavioural Drowsiness Score (BSD) is calculated using the following formula:

$$BDS=0.4\cdot \frac{2}{\pi }arctan(F_{blink})+0.3\cdot \frac{2}{\pi }arctan(F_{yawn})+0.3\cdot \frac{2}{\pi }arctan(F_{nod})$$

The video recording is analysed frame by frame. For each frame, facial landmarks are extracted using MediaPipe Face Mesh. Geometric features describing the eyes, mouth, and head pose are then computed. MediaPipe Face Mesh provides a dense set of mouth landmarks; however, only a selected subset of landmarks was used for EAR and MAE computations.

#### 3.2.1. EAR Computation

**Figure 3:**
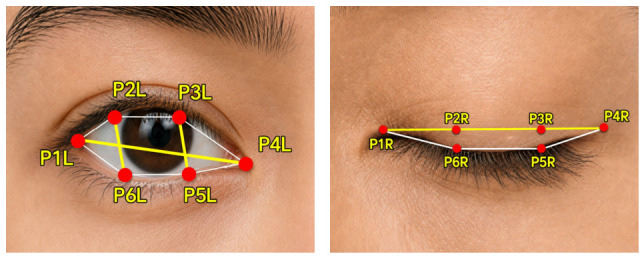
Eye feature landmarks.

As mentioned, MediaPipe Face Mesh returns a dense set of facial landmarks. However, EAR is not computed from the full mesh, but from a selected six-point subset around each eye. This preserves conceptual consistency with the classical definition of the EAR, in which vertical eye opening is normalised by eye width. The current implementation uses the landmarks shown in Figure 3.

For the left and right eye, EAR is computed using the following equations:

$$EAR_{\mathrm{left},i}=\frac{d\left(P_{2L,i},P_{6L,i}\right)+d\left(P_{3L,i},P_{5L,i}\right)}{2\cdot d\left(P_{1L,i},P_{4L,i}\right)}$$

$$EAR_{\mathrm{right},i}=\frac{d\left(P_{2R,i},P_{6R,i}\right)+d\left(P_{3R,i},P_{5R,i}\right)}{2\cdot d\left(P_{1R,i},P_{4R,i}\right)}$$

where $P_{1L},P_{2L},P_{3L},\;P_{4L},P_{5L},P_{6L},\;P_{1R},P_{2R},P_{3R},\;P_{4R},P_{5R},{\;andP}_{6R}$ denote semantically defined eye landmarks corresponding to MediaPipe indices 33, 160, 158, 133, 153, 144, 362, 385, 387, 263, 373 and 380, respectively.

In these equations, $P_{k,i}$ denotes the MediaPipe landmark with index k in frame i, and $d(P_{a,i},P_{b,i})$ denotes the Euclidean distance between points $P_{a,i}$ and $P_{b,i}$:

$$d(P_{a,i},P_{b,i})=\sqrt{{\left(x_{a,i}-x_{b,i}{)}^{2}+(y_{a,i}-y_{b,i}\right)}^{2}}$$

If normalised MediaPipe coordinates are used, computations can be performed directly in the normalised coordinate system or after rescaling to pixel coordinates. The key requirement is to preserve a consistent scale for all points within the same image.

The mean EAR for the frame is defined as

$$EAR_{i}=\frac{EAR_{left,i}+EAR_{right,i}}{2}$$

A low value of $EAR_{i}$ may indicate partially closed or closed eyes, but it is not by itself sufficient evidence of fatigue. EAR depends on facial geometry, camera angle, facial expression, eye asymmetry, landmark quality, and local deformation of the skin around the eyes. Therefore, in the subsequent stages, EAR is treated as an input signal for validation rather than as a direct indicator of driver state.

#### 3.2.2. MAR Computation

**Figure 4:**
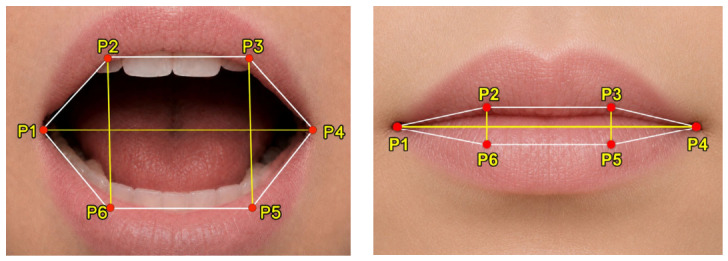
Mouth feature landmarks.

MAR is a geometric measure of mouth opening. As in the case of EAR, the computation uses a selected subset of MediaPipe landmarks rather than the full face mesh. In the current implementation, MAR is based on mouth width and two vertical distances describing the degree of mouth opening. The points selected for mouth opening determination are shown in Figure 4.

The following definition is used:

$$MAR_{i}=\frac{d(P_{3,i},P_{5,i})+d(P_{2,i},P_{6,i})}{2\cdot d(P_{1,i},P_{4,i})}$$

where $P_{1},P_{2},P_{3},\;P_{4},P_{5},{\;and\;P}_{6}$ denote semantically defined mouth landmarks corresponding to MediaPipe indices 78, 81, 311, 308, 402 and 178, respectively.

The Euclidian distance between points $P_{a,i}$ and $P_{b,i}$ is calculated in the same manner as described in Section 3.2.1 for EAR.

The MAR indicator is used in two different contexts. The first one concerns yawn detection, where a high mouth-opening threshold is applied. The second one regards the interpretation of low EAR in the happy facial-expression context. In that case, a lower mouth-activity threshold is used. This is described in more detail in Section 3.4.

#### 3.2.3. NOD Estimation

The $F_{nod}$ indicator is one of the components of the overall fatigue assessment. In the case of validation and happy-emotion context, the system primarily modifies eye-closure events and derived values such as PERCLOS and $F_{blink}$. The $F_{nod}$ value remains calculated from the pitch signal and detected head-drop episodes. The happy-context event filter, described later in Section 3.3, does not remove or modify nod events.

Conceptually, $F_{nod}$ acts as an independent information channel related to fatigue. Eye closures describe loss of alertness visible in the eyelid region, yawning describes mouth behaviour together with emotional context, and $F_{nod}$ describes a macroscopic change in head posture. Combining these signals reduces the dependence of the system on a single type of symptom. For example, a driver may show relatively few long eye closures but clear episodes of head dropping; in such a case, $F_{nod}$ increases the aggregated risk assessment.

Head position is estimated from a small set of facial landmarks. For a frame indexed by i, the method uses the left side point of the face $L_{i}$, the right side point of the face $R_{i}$, the nose point $N_{i}$, and the chin point $C_{i}$. In the implementation, these correspond to the following MediaPipe landmarks: $P_{234}$, $P_{454}$, $P_{1}$, and $P_{152}$. All coordinates are image coordinates, expressed in pixels after scaling from MediaPipe’s normalized landmark coordinates.

The face width is calculated first: $W_{i}=∥R_{i}-L_{i}∥+\varepsilon$, where ε is a small constant used to avoid division by zero. The midpoint of the face between the two lateral face points is then calculated as

$$M_{i}=\frac{L_{i}+R_{i}}{2}$$

The primary vertical component of the pitch signal is computed as the normalized vertical displacement of the nose relative to the face midpoint:

$$pitch_{i}^{\left(1\right)}=-100\cdot \frac{y_{N,i}-y_{M,i}}{W_{i}}$$

The negative sign follows from the image coordinate system, where the y-axis increases downward. As a result, the change in nose position relative to the geometric midpoint of the face produces a signal whose direction is consistent with the later logic for detecting head dropping. The value is also multiplied by 100 to obtain a convenient percentage-like scale similar to that of the other signals stored in the report.

A correction term based on the relationship between the nose and chin is then added. First, an approximate face height is calculated: $H_{i}=∥C_{i}-M_{i}∥+\varepsilon$.

The correction component is

$$pitch_{i}^{\left(2\right)}=10\cdot \frac{y_{C,i}-y_{N,i}}{H_{i}}$$

The final simplified pitch signal stored for the frame is $pitch_{i}=pitch_{i}^{\left(1\right)}+pitch_{i}^{\left(2\right)}$.

The value $pitch_{i}$, defined in this way, should not be interpreted as a physical head rotation angle. It is a relative indicator that depends on facial geometry in the image, camera position, landmark detection quality, and the distance between the face and the camera. Its advantage is its simplicity and sufficient stability for detecting longer, clearly visible head drops relative to the typical head position in a given video.

In the same path, a simplified roll signal is also calculated:

$$roll_{i}=100\cdot \frac{y_{R,i}-y_{L,i}}{W_{i}}$$

After the video has been processed, the $pitch_{i}$ values stored for consecutive frames are analysed again during construction of the driver-status table and the output report. Because different videos may have different frame geometries, camera placements, and natural head postures of the recorded subject, the pitch signal is centred around the median value computed over the entire video:

$$\tilde{p}=median(pitch_{1},pitch_{2},\ldots ,pitch_{N})$$

where N denotes the number of frames for which a pitch value is available. A centred signal is then calculated for each frame: $p_{i}^{\prime }=pitch_{i}-\tilde{p}$.

This centring prevents the algorithm from interpreting the absolute head position as a universal value shared by all subjects and recordings. Instead, the method evaluates deviations from the typical head position within the analysed video. This is particularly important when the camera is positioned at a different angle, the face is slightly rotated, or the person naturally sits with the head positioned higher or lower.

A drowsy nod event is defined as a continuous video segment of sufficient duration in which the centred pitch signal falls below the configured head-drop threshold. A single frame with a low pitch value is therefore not enough. The algorithm requires the threshold crossing to persist for a minimum number of frames determined by the video frame rate and the nod-duration parameter.

Let the head-drop threshold be denoted as $T_{pitch,nod}$. Its default value is negative, because the method detects a downward deviation of the pitch signal below the typical head position. For each frame, a binary variable is defined as

$$z_{i}=\{\begin{array}{cc} 1, & \mathrm{if}\;p_{i}^{\prime }<T_{pitch,nod}\\ 0, & \mathrm{otherwise} \end{array}$$

The minimum event length in frames is calculated as $N_{min,nod}=\left\lfloor FPS|T_{nod}\right\rfloor$, where FPS is the frame rate of the analysed video, and $T_{nod}$ is the minimum duration of the event in seconds, whose default value is 0.5 s.

An event $E_{j}$ is considered a drowsy head drop if there exists a continuous interval of frames

$$E_{j}=\{s_{j},s_{j}+1,\ldots ,e_{j}\}$$

for which

$$z_{i}=1\;\mathrm{for}\;\mathrm{every}\;i\in E_{j}$$

and

$$|E_{j}|=e_{j}-s_{j}+1\geq N_{min,nod}$$

The number of such intervals in the video is stored as the Number of Drowsy Nods, $N_{nod}$. The $F_{nod}$ indicator is the frequency of detected drowsy head-drop episodes over the duration of the video. First, the duration of the analysed recording is calculated:

$$D=\frac{N_{frames}}{FPS}$$

where $N_{frames}$ is the number of processed video frames. The number of detected nod events is then divided by the video duration:

$$F_{nod}=\frac{N_{nod}}{D}$$

### 3.3. Adaptive Eye Closure Detection and PERCLOS

Video-based driver fatigue detection is often formulated as a classification problem based on facial video. In practice, many algorithms rely on geometric indicators, with EAR as a measure of eye openness, MAR as a measure of mouth opening, PERCLOS as the percentage of time with closed or partially closed eyes, and head-pose signals such as pitch and roll. EAR is particularly important because low EAR is often directly interpreted as eye closure. MAR is mainly used for yawning detection, whereas PERCLOS aggregates the duration of eye closure over the whole video or over a time window. These indicators are useful but not semantic. Low EAR may correspond to fatigue-related eyelid closure, but it may also result from smiling, speech, squinting, glasses, reflections, camera angle or landmark uncertainty. Similarly, high MAR may indicate yawning, but also laughter, speech or other mouth-opening events. Therefore, driver-state classification requires contextual validation.

Publicly available datasets are essential for method comparison, but their interpretation requires caution. In datasets containing simulated drowsiness, the participant often expresses the following symptoms: opening the mouth widely, imitating yawning or producing exaggerated gestures. Such behaviours can be visually salient, but they do not necessarily reproduce the natural coordination of mouth, eyes, eyebrows and head observed during real fatigue.

Yawning is especially problematic. In many drowsy samples, high-MAR episodes are not accompanied by a decrease in EAR. The eyes remain open or even become more open than in the neutral condition. This pattern is problematic because a model may learn acted mouth opening rather than the state of fatigue.

In several analysed recordings, the same subject may exhibit a higher average EAR in the drowsy sequence than in the not-drowsy sequence. If EAR is interpreted as a proxy for eyelid closure, this observation indicates a mismatch between the class label and the facial geometry.

The consequence is a risk of shortcut learning. If drowsy samples systematically contain exaggerated yawning gestures and not-drowsy samples do not, a classifier may achieve high within-dataset accuracy by learning a dataset artifact. The benchmark score remains high, but the ability to generalize to natural driving conditions may be limited.

**Figure 5:**
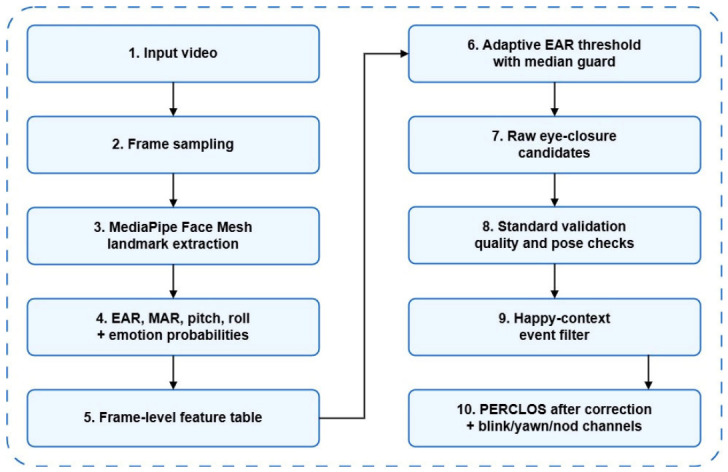
Workflow of the adaptive happy-context PERCLOS correction.

The overall workflow is shown in Figure 5. For each analysed frame, the system stores frame, time_s, left_ear, right_ear, ear, mar, pitch, roll, and emotion score from the model p_Neutral, p_Happy, p_Sad, p_Fear, p_Angry and emo_top, which is the emotion with the highest score. This frame-level table shows the interface between image processing and event analysis. It also enables diagnostics because it shows the context in which low-EAR frames occurred.

For adaptive EAR detection, the baseline eye-closure threshold is first determined:

$$T_{base}=r_{auto}\cdot {EAR}_{open\_baseline}$$

where ${EAR}_{open\_baseline}$ denotes the estimated EAR value for an open eye, and $r_{auto}$ is a scaling coefficient. By default, $r_{auto}=0.70$.

In practice, the baseline threshold may be unstable if the EAR distribution in the video is distorted by facial expression, camera perspective, or detection errors. A threshold that is too high causes too many frames to be treated as eye closure. Therefore, a median guard was introduced:

$$T_{median}=\;r_{median}\cdot median({EAR}_{valid})$$

where $r_{median}=0.85$.

And ${EAR}_{valid}$ denotes the set of reliable EAR values used to estimate the median. The final adaptive threshold is defined as

$$T_{EAR}=min(T_{base},T_{median})$$

This mechanism acts as a safeguard against an excessive threshold increase. If the baseline threshold is lower than or equal to the median guard, it remains unchanged. If, however, the baseline threshold is too high relative to the typical EAR level in the video, it is reduced. As a result, the method limits the number of false eye-closure candidates in recordings with active facial expression or atypical facial geometry.

### 3.4. MAR Indicator and Two Threshold Levels

In the method, MAR has two separate functions, and therefore, two threshold levels are used. The first threshold is used for yawn detection: $T_{MAR,\;yawn}=0.27$. This threshold is relatively high because yawning is typically associated with wide mouth opening and corresponds to the parameter used for detecting yawn events.

The second threshold is used by the happy-context filter, $T_{MAR,\;happy}=0.12$. This threshold is lower because it is not intended to detect yawning, but to identify active mouth expression. Smiling or speaking may increase MAR but usually does not have to exceed the threshold typical of yawning. The lower threshold makes it possible to identify events in which low EAR co-occurs with active facial expression and therefore may be an artefact of smiling or speaking. Methodologically, this can be written as $T_{MAR,\;yawn}>\;T_{MAR,\;happy}$. The two thresholds are not interchangeable. The first describes strong mouth opening typical of yawning. The second describes moderate mouth activity used as a context for interpreting low EAR.

Due to the different datasets, we employ one with frontal view (front camera) and one with angled view (camera at certain angle). In a frontal view, frame *i* is a raw eye-closure candidate when ${candidate}_{i}=\;\left[{EAR}_{i}<\;T_{EAR}\right]$. The square brackets indicate that the condition returns a logical value. The value true indicates that the frame satisfies the low-EAR criterion. For angled views, the rule is extended by relative EAR decreases for the left and right eye:

$${candidate}_{i}=\;\left({EAR}_{i}<\;T_{EAR}\right)\;\vee \;\left({EAR}_{left,i}<0.70\cdot {EAR}_{left,baseline}\right)\vee \left({EAR}_{left,i}<0.70\cdot {EAR}_{right,baseline}\right)$$

The symbol ∨ denotes logical “or”. A candidate may therefore be generated based on mean EAR or based on a relative drop in one eye. This extension increases sensitivity in more difficult views, but it may also increase the number of false candidates. Therefore, a raw candidate is not yet a fatigue-related event. It is only the input to validation.

Candidate validation is used to separate potential eye closures from artefacts and to handle frames with unreliable landmark detection or unstable facial geometry caused by occlusion, head rotation, or temporary landmark-tracking failures. The method rejects, among others, frames associated with unstable landmarks, abrupt changes in EAR or MAR, abnormal head pose, eye asymmetry, and strong facial-expression context.

$$p_{\mathrm{Happy},i}\geq T_{p,\mathrm{Happy}}\wedge MAR_{i}\geq T_{MAR,\mathrm{yawn}}$$

where: $T_{p,\mathrm{Happy}}=0.60$.

The symbol ∧ denotes logical “and”. The condition is satisfied only when high happy probability and MAR exceeding the yawn threshold occur simultaneously. This rule mainly detects strong laughter or very wide mouth opening. It does not, however, cover moderate smiling or speaking, where MAR may be elevated but still below the yawn threshold.

After this validation, continuous events are created from the confirmed frames. Short events are treated as blinks, intermediate events as uncertain, and sufficiently long events as fatigue-related eye closures. Only the latter are passed to the happy-context filter.

The happy-context filter operates at the event level, not at the level of individual frames. This means that its decision unit is a complete eye-closure episode, described by its start, end, and number of frames. This is important because a single frame with an incorrect emotion classification should not remove an event. Only a dominant happy context over a larger part of the event may indicate that the event is a probable false positive.

Let $E_{j}$ denote the j-th validated eye-closure event before the happy-context filter:

$$E_{j}=\{s_{j},s_{j}+1,\ldots ,e_{j}\}$$

where $s_{j}$ is the first frame of the event, and $e_{j}$ is the last one. The number of frames in the event is

$$|E_{j}|=e_{j}-s_{j}+1$$

The symbol $|E_{j}|$ denotes the cardinality of the set $E_{j}$, i.e., the number of frames belonging to the event.

For each frame in the event, a happy context flag is defined as

$$h_{i}=\{\begin{array}{cc} 1, & emo\_top_{i}=Happy\vee p_{Happy,i}\geq T_{pHappy}\\ 0, & otherwise \end{array}$$

The expression $emo\_top_{i}=Happy\vee p_{Happy,i}\geq T_{pHappy}$ means that the frame is treated as belonging to the happy context if happy is the dominant class or if the probability of this class exceeds the threshold $T_{pHappy}$. The value $p_{Happy,i}$ itself is not a logical condition; the logical condition is the comparison $p_{Happy,i}\geq T_{pHappy}$.

Next, the proportion of happy frames in the event is computed:

$$R_{Happy,E_{j}}=\frac{\sum_{i\in E_{j}}h_{i}}{|E_{j}|}$$

The numerator denotes the number of frames in the event that satisfied the happy condition. The denominator denotes the total number of frames in the event. Thus, $R_{Happy,E_{j}}$ is the proportion of happy frames within one eye-closure episode.

The following mean values are also computed for the event:

$${\bar{p}}_{Happy,E_{j}}=\frac{1}{|E_{j}|}\sum_{i\in E_{j}}p_{Happy,i}$$

$${\bar{MAR}}_{E_{j}}=\frac{1}{|E_{j}|}\sum_{i\in E_{j}}MAR_{i}$$

The event is rejected if

$$R_{Happy,E_{j}}\geq T_{rHappy}\wedge \left({\bar{MAR}}_{E_{j}}\geq T_{MAR,happy}\vee {\bar{p}}_{Happy,E_{j}}\geq T_{pHappy}\right)$$

where $T_{rHappy}=0.50,T_{MAR,happy}=0.12,T_{pHappy}=0.60$.

This rule requires two elements. First, at least half of the event must have a happy context. Second, there must be additional evidence of active facial expression, i.e., elevated mean MAR or high mean happy probability. This prevents the filter from removing events only because the happy label appeared in a few frames.

The constant values were selected according to their functional role in the validation pipeline rather than by direct optimization of the final classification accuracy. The parameters $r_{auto}$ and $r_{median}$ stabilize the adaptive EAR threshold and limit false eye-closure candidates caused by facial geometry, expression changes, or temporary landmark instability. The threshold $T_{pHappy}$ defines a reliable happy-related context for event filtering. These parameters are not used independently, but within a multi-stage validation procedure combining temporal consistency, mouth activity, head pose, landmark reliability, and emotional context.

In the following notation, it is necessary to separate the number of frames from the number of events. PERCLOS is a frame-based measure, whereas F_blink in the current implementation is the frequency of eye-closure events.

Raw PERCLOS is defined as

$$PERCLOS_{raw}=\frac{N_{frames,raw}}{N_{total}}\cdot 100$$

where $N_{frames,raw}$ denotes the number of frames satisfying the raw eye-closure candidate condition.

After standard validation, but before the happy-context filter, we obtain:

$$PERCLOS_{validated,before}=\frac{N_{frames,validated,before}}{N_{total}}\cdot 100$$

where $N_{frames,validated,before}$ denotes the number of frames belonging to validated fatigue-related eye-closure events before the happy-context filter.

After the happy-context filter, the final value is

$$PERCLOS_{validated,after}=\frac{N_{frames,validated,after}}{N_{total}}\cdot 100$$

where $N_{frames,validated,after}$ denotes the number of frames belonging to eye-closure events retained after filtering.

In the current adaptive validated path, the F_blink indicator is computed after the happy-context filter, but not from the number of frames. It is computed from the number of retained fatigue-related eye-closure events.

Let $N_{events,eye,after}$ denote the number of validated eye-closure events that remain after the happy-context filter. The video duration in seconds is

$$D=\frac{N_{total}}{FPS}$$

Then

$$F_{blink}=\frac{N_{events,eye,after}}{D}$$

### 3.5. Emotional Feature Modelling

In the proposed framework, emotional information is not used to directly infer driver fatigue; rather, it provides contextual cues to help interpret facial dynamics. In this work, emotions are not interpreted as psychological states but as observable facial expression patterns extracted from image data and used solely to contextualize behavioural indicators.

Fatigue is associated with changes in affective state, encompassing both positive and negative emotions; however, these do not contribute equally. Negative emotions such as irritability or stress have been shown to directly accelerate the progression and intensity of fatigue, while positive affect plays a stabilizing role, partially buffering fatigue-related deterioration. This asymmetry makes negative emotional states particularly informative as contextual indicators in fatigue detection [36,37,38].

Classical fatigue indicators such as eye closure, mouth opening, head nodding and PERCLOS are ambiguous because similar geometric patterns can be caused by both fatigue-related behaviour and normal facial expressions such as smiling, speaking, or squinting. To address this limitation, the proposed framework introduces several complementary modifications, among which emotion-aware modelling plays a central role.

Unlike methods based on frame-level emotion classification, which are subject to high variability and prediction noise, the proposed approach models the temporal variability of emotion distributions through EES and their weighted contribution through EWS, providing a more robust representation of fatigue-related facial patterns.

An emotion recognition model is first applied at the frame level to estimate the probability distribution over facial expressions, which is subsequently used both to refine the interpretation of primary signals, such as EAR and MAR during event detection and validation, and to derive aggregated emotional descriptors, including the EES, reflecting the variability of the emotional state, and the EWS, capturing the relative contribution of predefined emotion categories to fatigue-related facial deformation patterns. These descriptors constitute a key novelty of the proposed approach, as they move beyond conventional frame-level emotion classification and explicitly address the impact of prediction noise and temporal instability.

These parameters enable behavioural signals to be modulated by emphasizing semantically consistent patterns (e.g., tension during yawning) and suppressing patterns that are more likely to be related to expressive activity (e.g., laughter). The main advantages of this approach are improved interpretability and robustness against false positives caused by facial mimicry or dataset bias.

**Figure 6:**
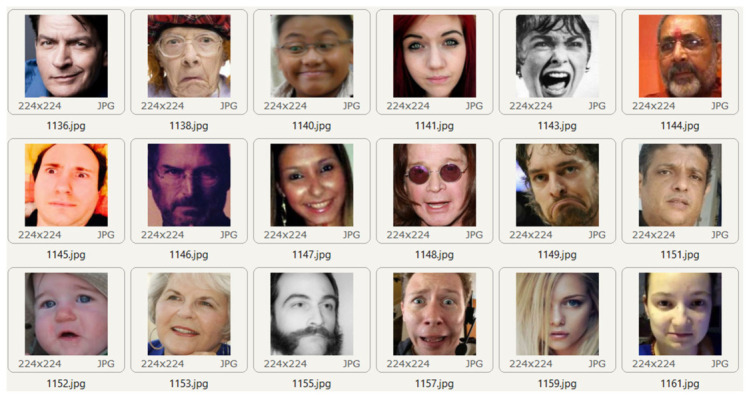
Sample images from AffectNet dataset.

To ensure the reliable estimation of these emotion distributions and their subsequent aggregation into EES and EWS, the underlying emotion recognition model was trained on a large-scale, real-life dataset. AffectNet [39] is a collected dataset comprising over one million facial images, with approximately ~440,000 samples manually annotated using both categorical emotion labels and continuous valence–arousal dimensions. In contrast to more constrained datasets, e.g., FER2013, it reflects substantial variability in real-world conditions, including pose, illumination, occlusions, and subject diversity, making it suitable for training robust emotion recognition models. AffectNet provides a richer annotation scheme (Figure 6), which improves the robustness of emotion recognition models when used as contextual information in driver fatigue detection. A notable limitation of AffectNet is its class imbalance, with a clear disparity between dominant categories representing positive or neutral expressions, such as happy (over 146,000 samples) and neutral (around 80,000), and its significant underrepresentation of negative emotions, including fear (≈8000), anger (≈28,130) and sadness (≈29,487).

EfficientNet-B0 was selected as a suitable backbone for facial emotion recognition due to its favourable balance between model complexity and representational capacity. While more complex architectures may achieve higher accuracy, the chosen model provides an efficient and sufficiently robust representation for the considered application. Moreover, ImageNet pretraining provides reliable low-level and mid-level visual features, ensuring good generalization to real-world facial data from the AffectNet dataset.

The network takes a 224 × 224 RGB image as input. Preprocessing involves rescaling and normalization, which are consistent with the ImageNet weights, to ensure that the pixel distribution aligns with the pretraining of the backbone. The backbone begins with a 3 × 3 convolution with 32 filters and a stride of 2, followed by a sequence of MBConv stages. Each MBConv block combines depthwise and pointwise convolutions with normalization and a nonlinear activation, allowing good feature quality with a relatively small number of parameters. Spatial resolution is reduced by convolutions with a stride of 2, meaning there are no separate max/average pooling layers between stages. For a 224 × 224 input, the tensor sizes evolve as follows: 112 × 112 × 32 → 56 × 56 × 24 → 28 × 28 × 40 → 14 × 14 × 80 → 14 × 14 × 112 → 7 × 7 × 192 → 7 × 7 × 320. The EfficientNet head concludes with a 1 × 1 convolution that produces a feature map of approximately 7 × 7 × 1280.

**Figure 7:**
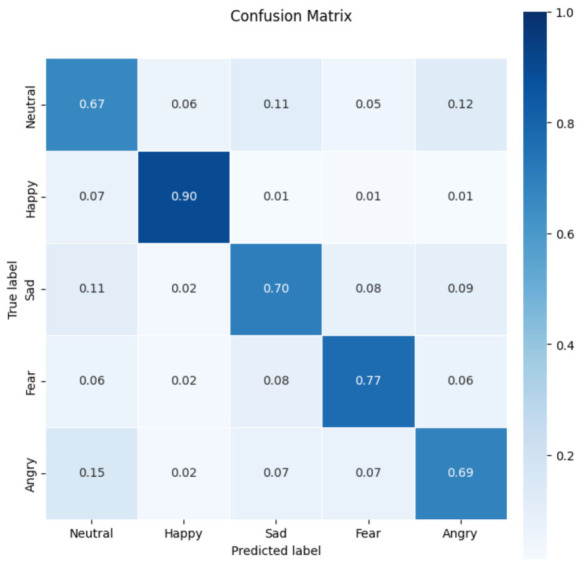
Confusion matrix for the five-class model.

The training procedure was performed in two stages. In the first stage, all layers of the EfficientNet backbone were frozen, and only the classification head was trained, allowing rapid adaptation to the target dataset while preserving generic visual features learned from ImageNet. In the second stage, the backbone was unfrozen, and the entire network was fine-tuned. Batch normalization layers were kept in inference mode to ensure stable statistics under limited batch size conditions. In the first phase, the model was optimized using the Adam optimizer with a learning rate of 5× and categorical cross-entropy loss. In the second phase, a lower learning rate of $1\times 10^{-5}$ was applied, together with label smoothing, to improve generalization. The learning rate followed a schedule consisting of a short warm-up phase followed by cosine decay. Early stopping and model checkpointing based on validation accuracy were employed in both stages. The resulting model performance is presented in Figure 7 using a confusion matrix.

The reduction of the emotion list to five classes in the suggested method comprises happy and neutral (corresponding to a positive or fatigue-neutral state), while sad, angry, and fearful relate to fatigue mechanisms. Such a modification adds more stability due to the minimization of the number of frames belonging to each emotion class and the avoidance of the influence of misclassification errors while preserving vital emotional data about fatigue. The model accuracy is presented in Table 1. Although the overall accuracy of the emotion recognition model only reaches 74%, it is sufficient for the proposed application, as emotional information is not used for direct classification but rather as a contextual signal that is further aggregated through EES and EWS, reducing the impact of individual prediction errors.

**Figure 8:**
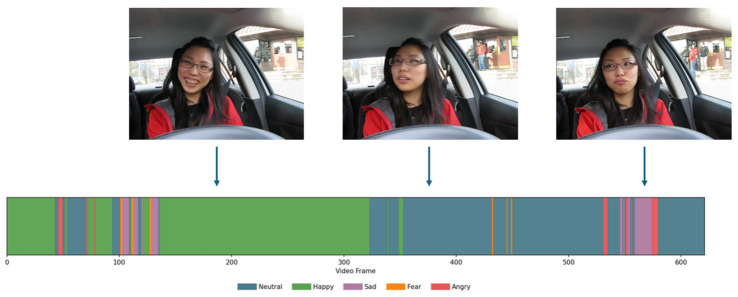
Sample frames and corresponding timeline of emotion predictions across the video sequence [13-FemaleGlasses, YawDD dataset].

An example of frame-level emotion predictions is shown in Figure 8, where the temporal distribution of predicted emotion classes illustrates the variability of instantaneous estimates. This representation corresponds to the initial stage of the proposed framework prior to the aggregation of emotional information into the EES and EWS descriptors. Such a representation facilitates intuitive visual analysis, as it reveals the temporal continuity of the emotional states, abrupt transitions, and potential misclassifications. For each frame, a vector of five probabilities $p_{i}$ is obtained, from which the dominant emotion is selected and visualized in the timeline (blue—neutral; green—happy; purple—sad; orange—fear; red—angry), providing a clear depiction of the model’s frame-level decisions.

For each frame *t*, the emotion recognition model outputs a probability vector:

$$p\left(t\right)=\left[p_{1}\left(t\right){,p}_{2}\left(t\right),\;{\ldots ,\;p}_{K}\left(t\right)\right],\;\sum_{i=1}^{K}p_{i}\left(t\right)=1$$

where each component corresponds to a specific emotion class. Next, the dominant emotion is first identified as the class with the highest probability:

$$i_{dominant}\left(t\right)=\arg\underset{i}{max}p_{i}\left(t\right)$$

This discrete representation allows for tracking of the evolution of emotional states over time. Next, within a temporal window *W*, the relative frequency of each emotion class is computed as

$$f_{i}=\frac{1}{N}\sum_{k\in W}1(i_{dominant}(k)=i)$$

where *N* denotes the number of frames in the window. This results in an empirical distribution of dominant emotions describing the temporal structure of affective behaviour.

Finally, the Emotional Entropy Score is defined using the normalized Shannon entropy:

$$EES\left(t\right)=-\frac{1}{\log K}\sum_{i=1}^{K}f_{i}\log f_{i}$$

which quantifies the variability of emotional states within the analysed window. Low EES values indicate a stable, dominant emotional pattern, corresponding to consistent facial behaviour and providing reliable conditions for fatigue detection. Conversely, high EES values reflect frequent emotional transitions, which are usually associated with expressive activities such as speaking, reacting or laughing. In such cases, fatigue-related indicators become less reliable. The normalized range of EES lies within [0|1], where values close to 0 indicate a single dominant emotion within the analyzed window, while values close to 1 correspond to high variability and frequent transitions between emotional states. The EES quantifies the temporal variability and stability of emotional patterns.

The EES provides information about the temporal variability of emotional states; however, it does not distinguish between fatigue-related and expression-driven fluctuations. The proposed approach addresses this by introducing the Emotion Weighted Score (EWS), which is defined as follows:

$$EWS\left(t\right)=\sum_{i=1}^{K}w_{i}p_{i}(t)$$

The EWS captures the semantic relevance of emotional states with respect to fatigue by assigning different weights to individual emotion classes and emphasizing those more strongly associated with fatigue-related behaviour. As a result, persistent negative emotions such as sadness, anger, and fear contribute more significantly to the final fatigue assessment, whereas neutral and positive states have a limited impact. In this way, EWS transforms raw emotional information into a fatigue-oriented representation, enabling the model to distinguish between emotionally stable patterns that are relevant to fatigue and those arising from non-fatigue-related facial activity.

The weighting scheme used in EWS (Table 2) reflects the different emotional states’ asymmetric contribution to fatigue-related processes. Positive and neutral emotions are given relatively low weights because they are not usually directly associated with fatigue and may correspond to normal driving conditions. In contrast, negative emotions such as sadness, fear and anger are assigned higher weights, reflecting their stronger association with fatigue, reduced cognitive control and increased emotional burden. Importantly, this weighting scheme is based on the semantic interpretation of emotions rather than dataset-specific distributions, meaning it is independent of the dataset.

### 3.6. Behavioural–Emotional Fusion and Driver Fatigue Risk Classification (BEFI, DFRI)

The final fatigue assessment is obtained through a two-stage process combining multimodal fusion and rule-based risk classification. We propose the Behavioural–Emotional Fatigue Index (BEFI) and the Driver Fatigue Risk Index (DFRI), which operate at different levels of interpretation. The BEFI performs a fusion of behavioural and emotional information by combining classical fatigue indicators (BDS) with emotional context through EWS and EES. In this formulation, behavioural fatigue signals are amplified when supported by semantically relevant and temporally stable emotional patterns, providing a context-aware estimate of fatigue intensity.

BEFI(t)=BDS(t)·(1+α·EWS(t)·(1−EES(t)))

The parameter α regulates the contribution of emotional information. The value α=3  was empirically selected as a balanced setting that highlights the influence of emotions without dominating the behavioural component; moreover, was not tuned to maximize accuracy on a specific dataset, but rather chosen to ensure a stable and interpretable contribution of emotional information across different conditions.

Finally, the proposed the Driver Fatigue Risk Index (DFRI) represents the final decision-level metric, integrating BEFI with eye-closure-based information (PERCLOS). This enables the simultaneous consideration of physiological and contextual factors, resulting in a more robust and interpretable assessment of driver fatigue risk. While the BEFI models the underlying fatigue mechanisms, the DFRI translates them into a practical risk indicator suitable for classification and decision making.

DFRI(t)=0.6·PERCLOS(t)+0.4·BEFI(t)

The weighting scheme in the DFRI reflects the complementary roles of physiological and behavioural–emotional indicators in fatigue detection. PERCLOS (0.6) is given a higher weighting, as eye-closure-based measures are well-established and directly linked to the physiological manifestations of fatigue. By contrast, the BEFI (0.4) incorporates behavioural and emotional cues to provide contextual information, enhancing interpretability but making it more sensitive to external factors.

Unlike conventional binary drowsy/non-drowsy approaches, which are often sensitive to dataset-specific characteristics and class distributions, the proposed DFRI formulation introduces a novel multi-level fatigue assessment with four distinct risk levels (Table 3), enabling a more expressive and interpretable characterization of driver state. The thresholds are dataset-independent and sufficiently separated to ensure stable and unambiguous decisions. This design is supported by a deeper and more controlled analysis of behavioural, physiological, and emotional information, allowing for a more robust and generalizable assessment of driver fatigue.

The DFRI thresholds were defined as operational risk zones rather than dataset-optimized classification boundaries. Since the DFRI combines validated PERCLOS with behavioural–emotional context, its values should be interpreted as a gradual accumulation of fatigue-related evidence rather than as direct probabilities of drowsiness. The lowest interval, DFRI < 0.03, corresponds to recordings in which both validated eye-closure evidence and contextual behavioural–emotional indicators remain close to the background level. The range 0.03 ≤ DFRI < 0.05 was introduced as a conservative transition zone, representing cases with weak but observable fatigue-related evidence. Values between 0.05 and 0.1 indicate a stronger accumulation of validated behavioural or physiological fatigue cues and are therefore interpreted as high fatigue risk. Finally, DFRI ≥ 0.1 corresponds to cases in which the combined contribution of the validated PERCLOS and contextual BEFI becomes sufficiently high to indicate an unsafe driver state.

Importantly, these thresholds are not intended to represent externally annotated mild, moderate, or severe fatigue stages. Instead, they provide interpretable decision regions for the proposed DFRI formulation. The separation between adjacent intervals was chosen to reduce sensitivity to small fluctuations in the index and to preserve a meaningful distinction between safe, uncertain, elevated-risk, and unsafe driving states.

## 4. Results

### 4.1. Selected Datasets

To ensure a comprehensive evaluation, two complementary datasets were selected, as they offer distinct yet mutually beneficial characteristics that justify their joint use. The Yawning Detection Dataset (YawDD) offers a wide range of facial expressions and behavioural patterns, making it ideal for analysing variability in facial activity and the subtle cues associated with fatigue-related behaviours, such as yawning. In contrast, the NTHU-DDD dataset offers a more structured framework for model development and evaluation, enabling the reliable, standardized assessment of detection performance. Consequently, the YawDD is primarily used to study expression dynamics and generalization, while the NTHU-DDD is used to set a benchmark for supervised performance evaluation.

The YawDD dataset comprises 351 video recordings, divided into two subsets. The first subset includes 322 short clips, captured with a camera mounted beneath the rear-view mirror. Each of the 29 participants appears multiple times (typically 3–4 times) in different behavioural states, such as normal driving, speaking/singing and yawning. The second subset contains 29 longer recordings, one per subject, covering all behaviours in a single, continuous sequence. All videos are stored in AVI format (640 × 480, 30 fps) and lack frame-level annotations, making the dataset more suitable for analysing temporal behaviour than for strict supervised learning.

**Figure 9:**
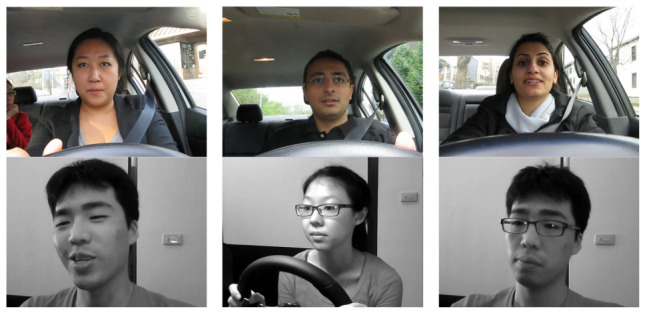
Sample frames from YawDD videos (**top**) and NTHU-DDD (**bottom**).

In contrast, the NTHU-DDD dataset is a more structured benchmark comprising 36 participants and approximately 9.5 h of recordings captured under diverse conditions (day and night, with or without glasses). These recordings are acquired at 30 fps during the day and 15 fps at night using infrared technology. They provide detailed annotations at both the frame and sequence levels (e.g., drowsiness, yawning and blinking), making them particularly well-suited to the supervised training of driver monitoring models. Figure 9 shows some sample frames from selected datasets.

### 4.2. Improving EAR/PERCLOS-Based Detection: Case Studies

Case #1—PERCLOS

The notdrowsy_person6 recording (based on NTHU-DDD) is a representative false-positive case. Visual assessment indicates an active subject who is smiling or speaking, without clear symptoms of drowsiness and without behaviour justifying a *Driving not recommended* classification. In the earlier variant, however, the result was dominated by PERCLOS because long low-EAR intervals were interpreted as eye closure.

The results (Table 4) show that the correction is not a blind suppression of the eye channel. In the segment where low EAR was not associated with a happy context, the filter did not remove the event. This is important because the method should reject only a well-defined false-positive mechanism, not every period of reduced eye openness.

Case #2—Adaptive EAR and Happy-Filter

The second case study concerns the person1 recording from the NTHU-DDD dataset, acquired with the camera positioned at an angle to the face. In the older dlib-based algorithm with a fixed EAR threshold, the subject was classified as drowsy and *Driving not recommended*. In the MediaPipe variant with an adaptive EAR threshold and happy-context PERCLOS correction, the result was classified as *Safe driving state*, which is consistent with the visual assessment.

**Figure 10:**
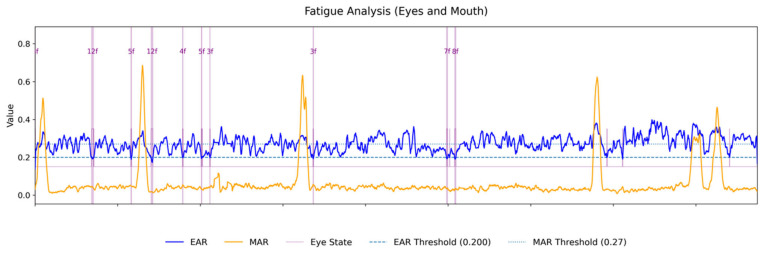
Analysis of person1 using dlib and a fixed EAR threshold. Under angled-view conditions, EAR interpretation is limited.

**Figure 11:**
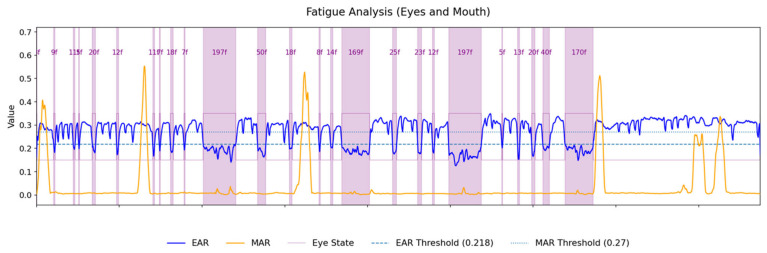
Analysis of person1 using MediaPipe and an adaptive EAR threshold. EAR drops are more temporally consistent with visual assessment but still require head-pose validation.

The comparison shows that landmark detector choice and thresholding strategy can substantially change the interpretation of the same recording. MediaPipe produces an EAR trajectory more consistent with visual assessment under non-frontal conditions (see Figure 10), whereas dlib may miss some eye-narrowing episodes or provide a less stable description when eye visibility is reduced (see Figure 11). At the same time, prolonged EAR decreases during downward head motion must be interpreted cautiously because they may result from actual eyelid closure or simply from reduced eye visibility.

Both case studies indicate that the problem is not EAR or PERCLOS themselves, but their overly direct interpretation. Low EAR should be analysed together with facial expression, MAR, head pose, landmark stability and temporal event structure. The adaptive variant reduces PERCLOS dominance when PERCLOS is inflated by smiling or speech.

The method is also diagnostic for datasets. If the drowsy class contains yawning with open eyes and the not-drowsy class contains smiling that reduces EAR, a simple algorithm may fail in both directions. An end-to-end model may still achieve high scores by learning dataset-specific gestures.

### 4.3. Evaluation of the Proposed Framework

**Figure 12:**
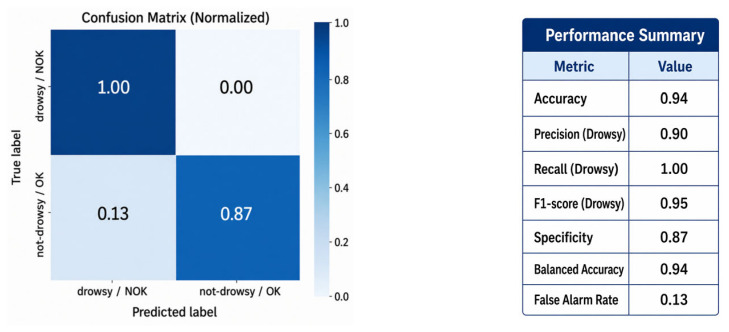
Normalized confusion matrix and performance summary for the binary driver-state classification.

The final DFRI-based decision was evaluated as a video-level classification output. For benchmark comparison, the four DFRI risk levels were mapped to two classes, i.e., *Safe driving state* and *Low fatigue risk* were treated as non-drowsy results, whereas *High fatigue risk* and *Driving not recommended* were treated as drowsy classifications. Based on this mapping, standard classification metrics were computed, including accuracy, recall, F1-score, and the confusion matrix. The precision, specificity, and balanced accuracy are also reported to provide a more complete description of the system behaviour (see Figure 12).

It should be noted that these metrics evaluate the final decision of the complete fatigue-detection pipeline and are not used as a trained deep-learning classifier. The proposed framework does not learn a drowsiness model from NTHU-DDD; instead, it produces a deterministic DFRI-based decision from fixed event-validation rules and predefined index formulations. Therefore, classification metrics are used here to quantify the agreement between the final system output and the dataset labels.

Since the proposed drowsiness assessment module is not trained on NTHU-DDD, conventional k-fold cross-validation is not directly applicable as a model-selection procedure. No frames or videos from NTHU-DDD are used to learn the decision function or to optimize classifier parameters. Nevertheless, subject dependency remains relevant for interpreting the results. Therefore, the evaluation is reported at the subject/video level, and frame-level random splits are avoided because they could artificially inflate performance due to temporal and subject-specific correlations.

Beyond the individual interpretation of the listed recordings, Table 5 provides a quantitative summary of the behaviour of the complete DFRI pipeline at the video level. The reported values include the main components of the proposed method, namely BDS; validated PERCLOS, EES, EWS, BEFI, and DFRI; and the final DFRI-based state. Therefore, the table should not be interpreted only as a set of isolated case analyses, but as a video-level quantitative evaluation of the complete fatigue-assessment procedure.

The results show a consistent separation between drowsy and non-drowsy recordings. All analysed drowsy sequences were assigned to elevated-risk categories: drowsy_person1 and drowsy_person3 were classified as Driving not recommended, while drowsy_person7 was classified as High fatigue risk. In contrast, the analysed non-drowsy sequences remained within the Safe driving state or Low fatigue risk regions, with notdrowsy_person1 and notdrowsy_person7 classified as in the Safe driving state and notdrowsy_person3 as at Low fatigue risk. This indicates that the combined DFRI preserves sensitivity to fatigue-related patterns when validated eye-closure information, behavioural frequencies, and emotion-based descriptors jointly support the drowsy interpretation, while reducing the risk of interpreting ambiguous facial activity as fatigue.

Importantly, this behaviour is maintained despite the presence of non-frontal views and pose-related landmark variability because low-EAR intervals are not directly converted into fatigue decisions. Instead, they are first subjected to adaptive thresholding, temporal event validation, head-pose-related consistency checks, and contextual filtering. The intermediate result for notdrowsy_person3 further shows that the proposed DFRI formulation does not force a binary interpretation, but expresses uncertainty through an interpretable low-risk state. Overall, these results support the usefulness of event-level validation and emotion-aware modulation for robust video-level driver-state assessment in observed drowsiness datasets. To further clarify the contribution of the main components of the proposed framework, a targeted component-level ablation-style analysis was added based on the previously reported video-level outputs of the complete pipeline.

### 4.4. Component-Level and Parameter-Role Analysis

Since the proposed method is a deterministic event-based framework rather than a trained classifier optimized on NTHU-DDD, the ablation was not formulated as a hyperparameter search. Instead, selected components were removed or simplified analytically from the reported BDS, PERCLOS, EES, EWS, BEFI, and DFRI values. The following variants were considered:

- Full proposed method—the original DFRI formulation.
- No emotional modulation—the emotional contribution was removed by setting BEFI = BDS, which corresponds to eliminating both EES and EWS from the fusion stage.
- No EES damping—the temporal-stability term was removed, resulting in the BEFI. This variant preserves the semantic emotional weighting but ignores whether the emotional pattern is temporally stable or unstable.
- BEFI only—the PERCLOS branch was removed, and only the behavioural–emotional component was retained as the diagnostic score.
- PERCLOS only—the behavioural–emotional branch was removed, and the diagnostic score was based only on validated PERCLOS.

The same binary interpretation threshold was retained for diagnostic comparison: videos with scores corresponding to High fatigue risk or Driving not recommended were treated as drowsy, while Safe driving state and Low fatigue risk were treated as non-drowsy. This corresponds to the DFRI threshold of 0.05 used in the proposed risk-level interpretation. No thresholds were re-optimized in any ablation variant.

The ablation results show that the full proposed formulation preserves the separation between drowsy and non-drowsy recordings in the analysed subset (see Table 6). Removing the emotional modulation decreases the score for all recordings, but the effect is particularly important for drowsy_person7, where the diagnostic value decreases from 0.0597 to 0.0417. Under the adopted DFRI threshold, this recording would no longer be assigned to the drowsy class. This indicates that the EES/EWS-based modulation contributes to preserving sensitivity in cases where PERCLOS alone is relatively moderate, but behavioural–emotional evidence supports fatigue-related interpretation.

Removing the EES damping increases the diagnostic score in all recordings, including non-drowsy samples. This confirms that EES acts as a stabilizing term that limits the influence of emotionally variable or transient facial activity. The effect is especially visible for notdrowsy_person7, where the score increases from 0.0126 to 0.0194 when EES damping is removed. Although this does not change the final binary decision in this subset, it shows that EES reduces unnecessary amplification of behavioural evidence in emotionally unstable non-drowsy recordings.

The BEFI-only and PERCLOS-only variants should be interpreted diagnostically because their values are no longer equivalent to the original DFRI scale. Nevertheless, they illustrate the complementary role of the two information streams. PERCLOS-only produces high values for drowsy_person1 and drowsy_person3, where eye-closure evidence is strong, but BEFI-only remains important for drowsy_person7, where the behavioural-emotional component supports the high-risk interpretation. These findings support the use of the DFRI as a fusion index rather than relying on a single information channel.

In particular, the decrease observed in the no-emotion variant for drowsy_person7 shows that emotional modulation can preserve sensitivity when PERCLOS evidence alone is not dominant. Conversely, the no-EES-damping variant shows that temporal emotional stability is useful for limiting score amplification in non-drowsy recordings.

The effect of the happy-context event filter is illustrated by the representative false-positive case discussed in Section 4.2 rather than treated as a separate dataset-wide ablation. In this case, low-EAR intervals were strongly associated with expressive facial activity, as indicated by the high proportion of happy-context frames and elevated mean MAR. After event-level filtering, PERCLOS decreased from 53.03% to 3.13%, and 18 events corresponding to 2026 frames and 67.53 s were removed. This supports the functional role of the filter as a mechanism for suppressing expression-related PERCLOS inflation.

It should also be noted that this analysis was not intended as an exhaustive sensitivity study of all threshold combinations. The main thresholds in the proposed framework have different functional roles and are used as operational gates within a multi-stage validation procedure rather than as independently optimized classifier parameters. In particular, the EAR threshold is estimated adaptively from the analysed sequence and constrained by the median guard, while the two MAR thresholds are used for different purposes, i.e., yawning-like wide mouth opening and moderate mouth activity in the happy-context filter. Similarly, the DFRI thresholds define operational risk zones rather than dataset-optimized class boundaries. Therefore, a full grid-based sensitivity analysis over all thresholds using the same acted-drowsiness benchmark could introduce dataset-specific tuning, which the proposed framework intentionally avoids. The present section should therefore be interpreted as a targeted component-level ablation and parameter-role analysis, while full multi-dataset sensitivity analysis remains an important direction for future work.

The main methodological implication is that reported high accuracies using public driver-drowsiness datasets should be interpreted together with an assessment of dataset realism, behavioural consistency and cross-dataset generalization. A dataset can be useful, but it should not be treated as an unproblematic substitute for natural driving conditions.

The proposed method is a step toward more critical validation. Including emotions does not mean psychologically interpreting the driver’s mood. Emotion classes are used as indicators of facial deformation: happy helps identify smiling or laughter, while fearful, sad and angry expressions may appear during the facial-muscle tension typical of yawning. Thus, high MAR with happy expressions can be treated as laughter, whereas high MAR with fearful/sad/angry expressions is more consistent with yawning.

Publicly available driver drowsiness datasets are essential for benchmarking, but their use requires careful interpretation. In particular, datasets containing acted or simulated drowsiness may include facial behaviours that are visually distinctive but not necessarily physiologically representative of real fatigue. As a result, models trained and evaluated on such data may achieve high classification accuracy while learning dataset-specific visual cues rather than comprise robust indicators of driver drowsiness.

In several analysed recordings, the same subject exhibited a higher average EAR in the drowsy condition than in the non-drowsy condition. This observation is counterintuitive if EAR is interpreted as a proxy for eyelid closure or reduced eye openness. The effect appears to be related to acted yawning episodes, during which the mouth is widely open but the eyes remain open or even become more open. Consequently, the drowsy label may be associated with exaggerated facial gestures rather than with consistent oculomotor signs of fatigue.

Adaptive PERCLOS validation with happy-context filtering improves the interpretability and robustness of the fatigue detector in cases where a visually alert subject is incorrectly classified as drowsy due to low EAR and high PERCLOS. The key response is to correct PERCLOS at the event level rather than to arbitrarily modifying the final score.

One of the goals of this study is to demonstrate that high benchmark performance on acted drowsiness datasets should not be directly interpreted as reliable real-world drowsiness detection. Dataset quality, behavioural naturalness, and label validity should be treated as critical factors in the evaluation of fatigue detection algorithms.

The analysed examples suggest that the drowsy class may contain visually salient but behaviourally ambiguous cues. In particular, acted yawning episodes often produce high MAR values, while EAR remains high or increases. Such a pattern differs from the expected coupling between yawning and partial eye closure observed in natural fatigue-related behaviour. Therefore, high classification accuracy obtained on this type of material may reflect the detection of acted mouth-opening gestures rather than robust fatigue recognition.

**Figure 13:**
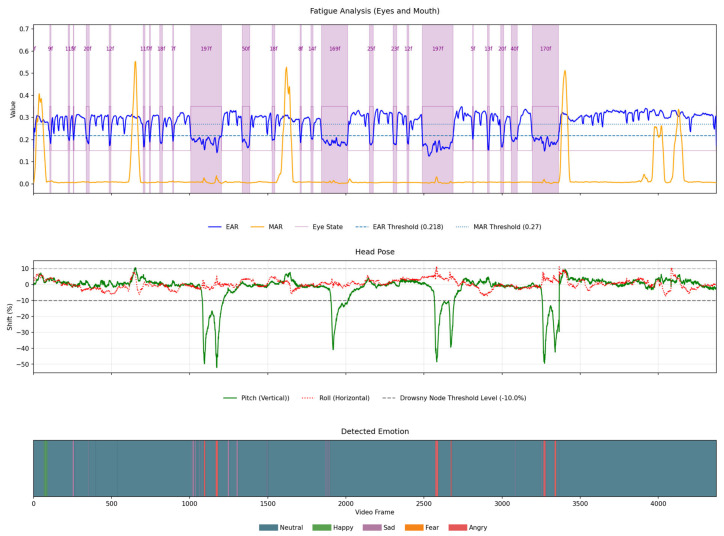
Summary of analysis for drowsy_person1.

**Figure 14:**
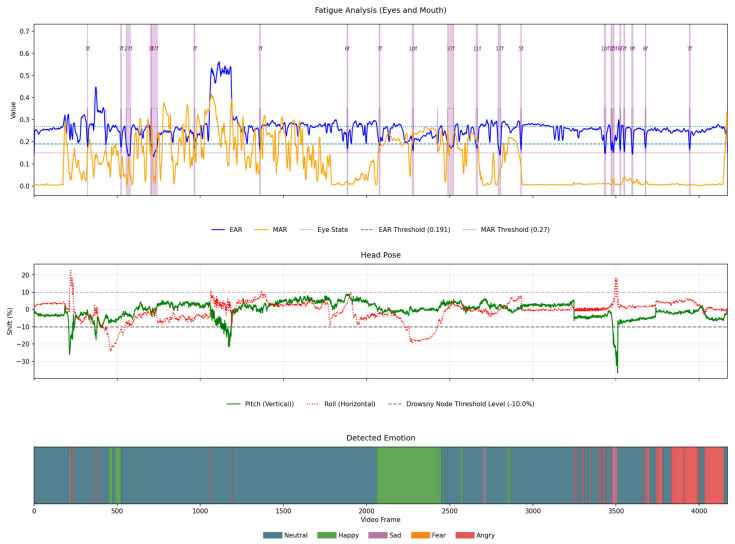
Summary of analysis for notdrowsy_person3.

Figure 13 and Figure 14 present a temporal summary of the proposed pipeline for representative drowsy and non-drowsy recordings, integrating eye-related, mouth-related, head-pose, and emotional signals over time. These visualizations are intended not only to illustrate individual examples, but also to show how the proposed method transforms heterogeneous frame-level measurements into temporally coherent behavioural patterns before computing the BEFI and DFRI.

In the drowsy case (Figure 13), the analysis reveals frequent and prolonged low-EAR intervals that remain consistent after adaptive validation, indicating that they are not merely isolated frame-level fluctuations. These eye-related events are accompanied by increased behavioural activity, distinct head-drop episodes, and emotion-weighted segments that support the interpretation of fatigue-related behaviour. The simultaneous occurrence of validated eye-closure evidence, behavioural frequency changes, head-pose deviations, and fatigue-relevant emotional descriptors results in a higher BEFI and consequently, an elevated DFRI value.

In contrast, the non-drowsy case (Figure 14) is characterized by more stable EAR trajectories, sparse and short-duration mouth activity, and the absence of sustained head-pose deviations. Although occasional transient events are visible, they do not form persistent temporal patterns and are therefore not strongly propagated to the final risk index. This illustrates the role of adaptive thresholding, event-level validation, and context-aware filtering in preventing short-term artefacts, normal facial expressions, or pose-related signal disturbances from being directly interpreted as fatigue.

Overall, Figure 13 and Figure 14 demonstrate that the proposed framework does not rely on single-frame decisions or isolated geometric thresholds. Instead, raw EAR, MAR, head-pose, and emotional signals are first evaluated in their temporal and contextual structure, and only validated evidence contributes to the BEFI and DFRI. This supports a clearer distinction between fatigue-related behaviour and normal driver activity, particularly in recordings containing expressive facial behaviour or non-frontal pose variations.

Table 7 provides a literature-level contextual comparison with selected methods evaluated on NTHU-DDD. It should not be interpreted as a fully standardized benchmark comparison. The reported results were obtained from the respective publications and may differ in terms of subject splits, train/test protocols, input modalities, annotation usage, evaluation units, label definitions, and performance metrics. Therefore, the table is intended only to position the proposed method relative to previously reported NTHU-DDD-based results rather than to claim direct superiority over methods evaluated under different protocols. The contextual comparison in Table 7 indicates that the proposed emotion-aware event-validated DFRI method obtains benchmark-level performance within the range reported by selected NTHU-DDD-based approaches. However, because the compared studies may use different experimental protocols, the numerical values should not be interpreted as a strict ranking of methods. Although some previously reported methods achieve higher accuracy, their results should be interpreted cautiously, as NTHU-DDD contains acted drowsiness patterns that may promote dataset-specific shortcut learning. In contrast, the proposed framework explicitly addresses the ambiguity of facial cues by combining eye- and mouth-based features with emotion-aware event validation, happy-context filtering, BDS, BEFI, and the final DFRI. The reported 94% accuracy should therefore be understood as performance regarding a controlled simulated-drowsiness benchmark. It does not by itself demonstrate dataset-independent generalization. Rather, it shows that the proposed event-validated and emotion-aware formulation can maintain competitive benchmark performance while providing additional diagnostic information about ambiguous or potentially artefactual facial behaviour.

It should be emphasized that the use of NTHU-DDD in this study has two complementary roles. First, it provides a structured, publicly available, and widely adopted benchmark that enables reproducible comparison with previous work. Second, it serves as diagnostic material illustrating the limitations of direct interpretation of acted drowsiness labels. The criticism of acted drowsiness datasets is therefore not a rejection of their usefulness, but a motivation for cautious interpretation and for the proposed event-level validation mechanism. In particular, NTHU-DDD contains simulated behaviours that may be visually salient but not always physiologically consistent with natural fatigue, such as exaggerated yawning-like gestures or mouth opening without coordinated eyelid closure. For this reason, the reported NTHU-DDD result should be interpreted as a benchmark result under the specified protocol and not as proof of real-world robustness or full dataset-independent generalization.

## 5. Conclusions

This work investigated the limitations of widely used vision-based driver fatigue indicators, highlighting that metrics such as EAR and PERCLOS, when interpreted in isolation, may lead to misleading conclusions under realistic conditions. In particular, normal facial behaviours, including speech, smiling, or non-frontal head poses, can produce geometric patterns similar to fatigue symptoms, increasing the risk of false-positive detections. These observations underline the need for a more context-aware and structured interpretation of facial signals.

To address this issue, a unified framework integrating behavioural, geometric, and emotional information was proposed. The method introduces adaptive eye-closure detection combined with multi-stage validation and event-level modelling, allowing fatigue-related events to be distinguished from transient or expression-driven artefacts. A key element of the approach is the incorporation of emotional context, not as a direct indicator of driver state, but as a supporting signal that helps interpret ambiguous facial dynamics. This is achieved through the introduction of aggregated descriptors, EES and EWS, which capture both temporal variability and the semantic relevance of emotional patterns.

The fusion of behavioural and emotional cues within the BEFI, followed by their integration with PERCLOS in the DFRI formulation, enables a multi-level and interpretable representation of fatigue risk. Unlike conventional binary classification approaches, the proposed method provides a graded assessment that better reflects the continuous nature of fatigue development. The proposed formulation was not optimized solely to maximize performance on NTHU-DDD; however, full dataset-independent validation remains an open requirement. The current results demonstrate the feasibility and interpretability of the proposed approach on controlled and semi-controlled material, while future work must include systematic cross-dataset validation under different camera positions, illumination conditions, subject populations, and naturalistic fatigue scenarios.

The results demonstrate that incorporating contextual and temporal information leads to a more consistent interpretation of fatigue-related signals, particularly in cases where classical indicators may be distorted by non-fatigue-related factors. At the same time, the analysis highlights the influence of dataset characteristics on evaluation outcomes, suggesting that performance obtained on controlled or simulated data should be interpreted with caution.

Although the proposed framework provides an interpretable, event-based assessment of driver fatigue, future work will focus on evaluating the proposed method using other real-scenario driving datasets and establishing cross-dataset comparisons using unified evaluation protocols. Furthermore, future work will entail analysing and enhancing emotion recognition methods, alongside conducting a more in-depth investigation into the temporal dynamics of emotions and their relationship with fatigue progression. Furthermore, the proposed framework will be extended to incorporate driver distraction analysis, enabling a more comprehensive evaluation of the driver’s state. The authors encourage further research and collaboration in this area to support the development and validation of context-aware driver monitoring systems.

## Figures and Tables

**Table 1:** Accuracy for the emotion recognition model.

Emotion Class	Accuracy [%]
Neutral	63.0
Happy	88.0
Sad	72.0
Fearful	78.0
Angry	71.0
Overall accuracy	74.0

**Table 2:** Weighting scheme for emotion classes used in EWS.

Emotion Class	Weight
Happy	0.1
Neutral	0.3
Sad	0.8
Fearful	0.9
Angry	1.0

**Table 3:** Proposed operational fatigue-risk levels used for DFRI interpretation in the present evaluation.

Fatigue Risk Index	Weight
Safe driving state	DFRI < 0.03
Low fatigue risk	0.03 ≤ DFRI < 0.05
High fatigue risk	0.05 ≤ DFRI < 0.1
Driving not recommended	DFRI ≥ 0.1

**Table 4:** Results for Case #1.

Stage/Metric	Result	Interpretation
Previous interpretation	PERCLOS_validated about 46.76%	Driving not recommended
Adaptive variant	PERCLOS_raw 53.03%, PERCLOS after happy-context 3.13%	Safe driving state
Low-EAR diagnostics	92.65% low-EAR during happy context; mean p_Happy 0.8368; mean MAR 0.1752	Low EAR linked to facial expression
Happy-context filter	18 events, 2026 frames, 67.53 s rejected	Expression contribution removed from PERCLOS

**Table 5:** Driver status classification test for selected subjects.

Subject	BDS	PERCLOS (/100)	EES	EWS	BEFI	DFRI	DFRI State
drowsy_person1	0.0499	0.2019	0.1119	0.3157	0.0919	0.1579	Driving not recommended
drowsy_person3	0.0526	0.2728	0.5675	0.6463	0.0967	0.2024	Driving not recommended
drowsy_person7	0.0214	0.0553	0.1901	0.8643	0.0664	0.0597	High fatigue risk
notdrowsy_person1	0.0021	0.0000	0.3789	0.2402	0.0031	0.0012	Safe driving state
notdrowsy_person3	0.0229	0.0406	0.4361	0.3392	0.0360	0.0388	Low fatigue risk
notdrowsy_person7	0.0153	0.0039	0.6224	0.5936	0.0256	0.0126	Safe driving state

**Table 6:** Targeted ablation analysis based on the video-level values.

Subject	Full DFRI	No EES/EWS BEFI = BDS	No EES Damping	BEFI Only	PERCLOS Only
drowsy_person1	0.1579	0.1411	0.1600	0.0919	0.2019
drowsy_person3	0.2024	0.1847	0.2255	0.0967	0.2728
drowsy_person7	0.0597	0.0417	0.0639	0.0664	0.0553
notdrowsy_person1	0.0012	0.0008	0.0014	0.0031	0.0000
notdrowsy_person3	0.0388	0.0335	0.0428	0.0360	0.0406
notdrowsy_person7	0.0126	0.0085	0.0194	0.0256	0.0039

**Table 7:** Literature-level contextual comparison of the proposed emotion-aware event-validated DFRI method with selected NTHU-DDD-based driver drowsiness detection approaches.

Method	Year	Dataset	Feature	Algorithm	Accuracy
[40]	2019	NTHU-DDD	Eye and Mouth	Gamma fatigue detection network	97.06%
[41]	2019	NTHU-DDD	Eye, Mouth and Head	3D convolutional networks	76.2%
[8]	2020	NTHU-DDD	Eye, Mouth and Head	3D convolutional networks	92.19%
[42]	2021	NTHU-DDD	Eye, Mouth and Head	CNN	85%
[43]	2022	NTHU-DDD	Eye, Mouth and Head	RF, SVM, and Sequential NN	RF: 99%; SVM: 80%; Sequential NN: 96%
Proposed method	2026	NTHU-DDD	Eye, Mouth and Emotions	Emotion-aware event-validated DFRI	94%

## Data Availability

The data supporting the findings of this study are available from publicly accessible repositories. The research utilized the AffectNet dataset for emotion recognition, which can be obtained upon prior request at: http://mohammadmahoor.com/pages/databases/affectnet/ (accessed on 18 May 2026). For driver fatigue analysis, the Yawning Detection Dataset (YawDD) was used and is available at IEEE DataPort: https://ieee-dataport.org/open-access/yawdd-yawning-detection-dataset (accessed on 18 May 2026). Additionally, the NTHU Driver Drowsiness Detection Dataset (NTHU-DDD) is officially hosted by the NTHU CV Laboratory and is accessible at: http://cv.cs.nthu.edu.tw/php/callforpaper/datasets/DDD/ (accessed on 18 May 2026). No new datasets were generated during this study. For further information or inquiries, please contact the corresponding author.

## References

[1] Goldenbeld C., Nikolaou D. (2019). Driver Fatigue.

[2] Tefft B.C. (2024). Drowsy Driving in Fatal Crashes, United States, 2017–2021 (Research Brief).

[3] May J.F., Baldwin C.L. (2009). Driver Fatigue: The Importance of Identifying Causal Factors of Fatigue When Considering Detection and Countermeasure Technologies. Transp. Res. Part F Traffic Psychol. Behav..

[4] Zhang H., Ni D., Ding N., Sun Y., Zhang Q., Li X. (2023). Structural Analysis of Driver Fatigue Behavior: A Systematic Review. Transp. Res. Interdiscip. Perspect..

[5] Ramzan M., Khan H.U., Awan S.M., Ismail A., Ilyas M., Mahmood A. (2019). A Survey on State-of-the-Art Drowsiness Detection Techniques. IEEE Access.

[6] Albadawi Y., Takruri M., Awad M. (2022). A Review of Recent Developments in Driver Drowsiness Detection Systems. Sensors.

[7] Kolus A. (2024). A Systematic Review on Driver Drowsiness Detection Using Eye Activity Measures. IEEE Access.

[8] Ed-Doughmi Y., Idrissi N., Hbali Y. (2020). Real-Time System for Driver Fatigue Detection Based on a Recurrent Neuronal Network. J. Imaging.

[9] Huynh X.P., Park S.M., Kim Y.G. (2017). Detection of Driver Drowsiness Using 3D Deep Neural Network and Semi-Supervised Gradient Boosting Machine. Computer Vision—ACCV 2016 Workshops; Lecture Notes in Computer Science.

[10] Alonazi M., Alshahrani H.J., Alotaibi F.A., Maray M., Alghamdi M., Sayed A. (2023). Automated Facial Emotion Recognition Using the Pelican Optimization Algorithm with a Deep Convolutional Neural Network. Electronics.

[11] Kowalczuk Z., Czubenko M., Merta T. (2019). Emotion Monitoring System for Drivers. IFAC-PapersOnLine.

[12] Li W., Huang J., Xie G., Karray F., Li R. (2021). A Survey on Vision-Based Driver Distraction Analysis. J. Syst. Archit..

[13] Liu Y., Wang C., Lu M., Yang J., Gui J., Zhang S. (2024). From Simple to Complex Scenes: Learning Robust Feature Representations for Accurate Human Parsing. IEEE Trans. Pattern Anal. Mach. Intell..

[14] Wang S., Wang C., Shi C., Liu Y., Lu M. (2024). Mask-Guided Mamba Fusion for Drone-Based Visible-Infrared Vehicle Detection. IEEE Trans. Geosci. Remote Sens..

[15] Li R., Chen Y.V., Zhang L. (2021). A Method for Fatigue Detection Based on Driver’s Steering Wheel Grip. Int. J. Ind. Ergon..

[16] Lu J., Zheng X., Tang L., Zhang T., Sheng Q.Z., Wang C., Jin J., Yu S., Zhou W. (2021). Can Steering Wheel Detect Your Driving Fatigue?. IEEE Trans. Veh. Technol..

[17] Vempati R., Sharma L.D. (2023). A Systematic Review on Automated Human Emotion Recognition Using Electroencephalogram Signals and Artificial Intelligence. Results Eng..

[18] Gromer M., Salb D., Walzer T., Martínez Madrid N., Seepold R. (2019). ECG Sensor for Detection of Driver’s Drowsiness. Procedia Comput. Sci..

[19] Vaussenat F., Bhattacharya A., Payette J., Saidi A., Bellemin V., Renaud-Dumoulin G.G., Cloutier S.G., Gagnon G. (2026). Early Drowsiness Detection via Second-Order Derivative Analysis of Heart Rate Variability: A Non-Contact ECG Approach with Machine Learning. Sensors.

[20] Arefnezhad S., Hamet J., Eichberger A., Frühwirth M., Ischebeck A., Koglbauer I., Moser M., Yousefi A. (2022). Driver Drowsiness Estimation Using EEG Signals with a Dynamical Encoder–Decoder Modelling Framework. Sci. Rep..

[21] Cao S., Feng P., Kang W., Chen Z., Wang B. (2025). Optimized Driver Fatigue Detection Method Using Multimodal Neural Networks. Sci. Rep..

[22] Chandiwala J., Agarwal S. Driver’s Real-Time Drowsiness Detection Using Adaptable Eye Aspect Ratio and Smart Alarm System. Proceedings of the 2021 7th International Conference on Advanced Computing and Communication Systems (ICACCS).

[23] Moujahid A., Dornaika F., Arganda-Carreras I., Reta J. (2021). Efficient and Compact Face Descriptor for Driver Drowsiness Detection. Expert Syst. Appl..

[24] Alioua N., Amine A., Rziza M. (2014). Driver’s Fatigue Detection Based on Yawning Extraction. Int. J. Veh. Technol..

[25] Weng C.-H., Lai Y.-H., Lai S.-H. Driver Drowsiness Detection via a Hierarchical Temporal Deep Belief Network. Proceedings of the Asian Conference on Computer Vision Workshops.

[26] Abtahi S., Omidyeganeh M., Shirmohammadi S., Hariri B. YawDD: A Yawning Detection Dataset. Proceedings of the ACM Multimedia Systems Conference.

[27] Oghamudu A., Haythornthwaite C., Barshan B. Driver Drowsiness Detection Based on Joint Monitoring of Yawning, Blinking and Nodding. Proceedings of the IEEE International Conference on Systems, Man, and Cybernetics (SMC).

[28] Soukupová T., Čech J. Real-Time Eye Blink Detection Using Facial Landmarks. Proceedings of the IEEE Conference on Computer Vision and Pattern Recognition Workshops (CVPR Workshops).

[29] Azim T., Jaffar M.A., Ramzan M., Mirza A.M. (2009). Automatic Fatigue Detection of Drivers through Yawning Analysis. Proceedings of the International Conference on Signal Processing (SIP); Lecture Notes in Computer Science.

[30] Abtahi S., Hariri B., Shirmohammadi S. (2014). Driver Drowsiness Monitoring Based on Yawning Detection. Proceedings of the 2011 IEEE International Instrumentation and Measurement Technology Conference.

[31] Cichocka S., Rumiński J. Driver Fatigue Detection Method Based on Facial Image Analysis. Proceedings of the 2024 16th International Conference on Human System Interaction (HSI).

[32] You F., Li X., Gong Y., Wang H., Li H. (2019). A Real-Time Driving Drowsiness Detection Algorithm with Individual Differences Consideration. IEEE Access.

[33] Krishna G.S., Supriya K., Vardhan J. (2022). Vision Transformers and YOLOv5 Based Driver Drowsiness Detection Framework. arXiv.

[34] Ghoddoosian R., Galib M., Athitsos V. (2019). A Realistic Dataset and Baseline Temporal Model for Early Drowsiness Detection. Proceedings of the 2019 IEEE/CVF Conference on Computer Vision and Pattern Recognition Workshops (CVPRW).

[35] Du G., Li T., Li C., Liu P.X., Li D. (2021). Vision-Based Fatigue Driving Recognition Method Integrating Heart Rate and Facial Features. IEEE Trans. Intell. Transp. Syst..

[36] Chen L., Huang Y., Wang F., Zhu L., Liu Z. (2026). Exploring the Impact of Emotional States on Fatigue Evolution in Metro Drivers: A Physiological Signal-Based Approach. Appl. Sci..

[37] Tomaso C.C., Johnson A.B., Nelson T.D. (2021). The Effect of Sleep Deprivation and Restriction on Mood, Emotion, and Emotion Regulation: Three Meta-Analyses in One. Sleep.

[38] Shang Y., Yang M., Cui J., Cui L., Huang Z., Li X. (2023). Driver Emotion and Fatigue State Detection Based on Time Series Fusion. Electronics.

[39] Mollahosseini A., Hasani B., Mahoor M.H. (2019). AffectNet: A Database for Facial Expression, Valence, and Arousal Computing in the Wild. IEEE Trans. Affect. Comput..

[40] Liu W., Qian J., Yao Z., Jiao X., Pan J. (2019). Convolutional Two-Stream Network Using Multi-Facial Feature Fusion for Driver Fatigue Detection. Future Internet.

[41] Yu J., Park S., Lee S., Jeon M. (2019). Driver Drowsiness Detection Using Condition-Adaptive Representation Learning Framework. IEEE Trans. Intell. Transp. Syst..

[42] Dua M., Shakshi, Singla R., Raj S., Jangra A. (2021). Deep CNN Models-Based Ensemble Approach to Driver Drowsiness Detection. Neural Comput. Appl..

[43] Albadawi Y., AlRedhaei A., Takruri M. (2023). Real-Time Machine Learning-Based Driver Drowsiness Detection Using Visual Features. J. Imaging.

